# Predictors of autism spectrum disorder diagnosis in a spanish sample of preterm children with very low birthweight: A cross‐sectional study

**DOI:** 10.1002/hsr2.1143

**Published:** 2023-03-02

**Authors:** María Magán‐Maganto, Ricardo Canal‐Bedia, Álvaro Bejarano‐Martín, Maria Victoria Martín‐Cilleros, Aránzazu Hernández‐Fabián, Andrea Luz Calvarro‐Castañeda, Herbert Roeyers, Cristina Jenaro‐Río, Manuel Posada de la Paz

**Affiliations:** ^1^ University Institute of Community Integration (INICO), Centro de Atención Integral al Autismo (INFOAUTISMO), Faculty of Education Universidad de Salamanca Salamanca Spain; ^2^ Pediatrics, Hospital Universitario de Salamanca Universidad de Salamanca Salamanca Spain; ^3^ Department of Experimental Clinical and Health Psychology, Research in Developmental Disorders Lab (RIDDL) Ghent University Ghent Belgium; ^4^ University Institute of Community Integration (INICO), Faculty of Psychology Universidad de Salamanca Salamanca Spain; ^5^ Rare Diseases Institute of Rare Diseases Research & Institute of Health Carlos III Madrid Spain

**Keywords:** autistic spectrum disorder, epidemiology, neuropsychology, preterm birth

## Abstract

**Background and Aims:**

Autism spectrum disorder (ASD) is a neurodevelopmental disorder with a higher likelihood of being diagnosed in preterm populations. Likewise, low birthweight has also been connected with an increased likelihood of ASD. The objectives were to study the frequency and define the relationship between ASD, gestational age, birthweight, and growth percentiles for preterm children.

**Methods:**

A sample of preterm children with very low birthweight was selected from the Spanish population at 7–10 years old. Families were contacted from the hospital, and they were offered an appointment to conduct a neuropsychological assessment. The children who showed signs of ASD were referred to the diagnostic unit for differential diagnosis.

**Results:**

A total of 57 children completed full assessments, with 4 confirmed ASD diagnoses. The estimated prevalence was 7.02%. There were statistically significant weak correlations between ASD and gestational age (*τb* = −0.23), and birthweight (*τb* = −0.25), suggesting there is a higher likelihood of developing ASD for those born smaller or earlier in their gestation.

**Conclusion:**

These results could improve ASD detection and outcomes for this vulnerable population while also supporting and enhancing previous findings.

## 1 INTRODUCTION

Preterm birth refers to any birth before 37 weeks of gestation^1^ and is a key contributor to childhood morbidity.^2^ It is more common in low and middle‐income countries, with 5,9% of births being preterm in Spain in 2020.^3^ However, with improvements in neonatal care, the rate of survival for prematurely born children has increased.^4^ Although there have been advances to reduce the incidence of problems associated with premature birth, there is still a higher risk of physical and neurodevelopmental disorders co‐occurring for this population, such as behavioral and learning difficulties and deficits in executive function.^5^

Another factor that has been associated with neurologic disability and diminished language development is low birthweight (BW).^6^ Very low birthweight (VLBW < 1500 g) or extremely low birthweight (ELBW < 1000 g)^7^ are often the result of preterm birth or some form of growth restriction within the uterus. In fact, the combination of low gestational age (GA) and low BW has been shown to lead to a heightened risk of many physical and developmental problems.^8^

Growth percentiles (GPs) are a way to view the measures of GA and BW together. Newborns who are small for gestational age (SGA), meaning lower than the 10th centile,^9^ have not only more risk to be born preterm, but also to develop more neonatal health difficulties in the long term.^10^ In recent meta‐analyses, preterm birth was associated with a larger variety of neurodevelopmental trajectories,^11^ while SGA and slow growth rate could lead to cognitive impairment through childhood.^12^ Measures to estimate fetal and postnatal growth are still contentious regarding what factors better define growth curves in different populations (see Cordova & Belford^13^ and Easter^10^ for more details regarding preterm children).

Autism spectrum disorder (ASD) is a neurodevelopmental disorder whose symptoms usually appear in early childhood and is characterized by difficulties in communication and social interaction, in addition to presenting repetitive behaviors, stereotyped interests, and unusual sensory behaviors that affect many areas of the daily life.^14^ ASD has been linked with preterm birth, with a recent meta‐analysis reporting a preterm ASD prevalence of 7% (one in 14; confidence interval [CI] = 4%–9%),^15^ which is higher than a prevalence of 2.3% (one in 44) found in the general population.^16^, ^17^ This difference suggests there is a higher prevalence of ASD in the population with premature birth. Hence, further study of the expression of ASD in preterm birth samples can be seen as an important contribution to this field.

It is important to note that the probability of an ASD diagnosis is often calculated for the entire preterm population. For example, a subject who was born with a GA of 36 weeks and another with a GA of 25 weeks would be treated as part of the same group to be compared to the general population or other groups as categorical data (see Agrawal et al.^15^ and Wang et al.^18^). However, there is the possibility that a child with lower GA, BW or GP, within the preterm population, would have a higher likelihood to develop ASD.

Given the results of the recent meta‐analysis,^15^ GA, measured in weeks, was hypothesized to be a potential risk factor of ASD. Alternatively, BW, which is highly associated with GA, might be a better way to estimate ASD risk, as it can be measured more accurately than GA and could better represent the actual development of the child. It was thought that GP may also be a good predictor of ASD risk, where a lower GP would mean higher risk. Up until now, there has not been any study in Spain that has analyzed the prevalence in premature populations or evaluated the link between ASD and BW, GA, or GP.

Therefore, the main objective of this research was to study the frequency of ASD in a preterm birth and VLBW sample of children born between 2009 and 2011 in the northwest of Spain. This included gaining a better understanding of the relationship between ASD and GA, BW, or GP.

## 2 METHODS

### 2.1 Case ascertainment

**Figure 1:**
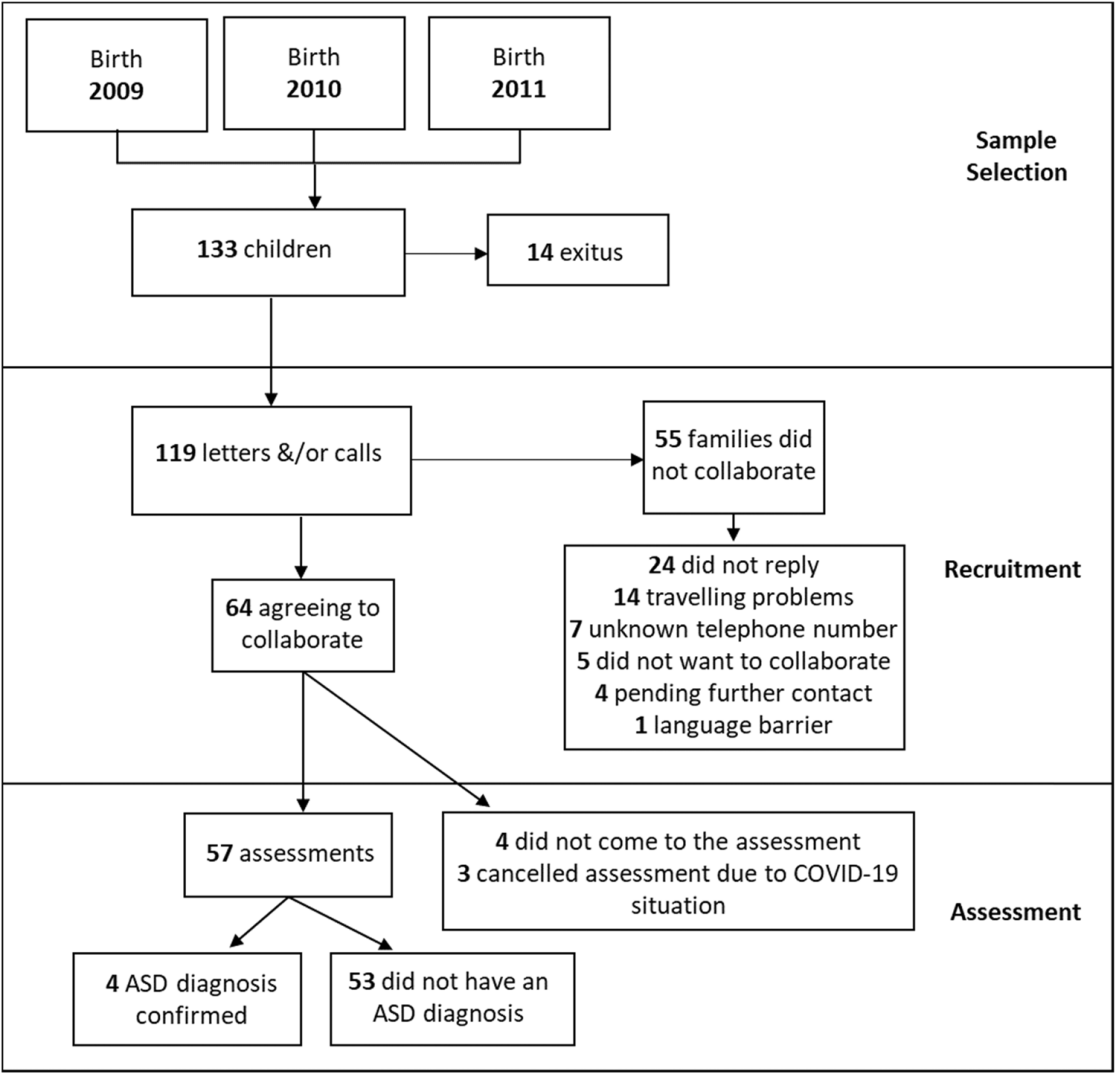
Sample selection, recruitment, and assessment flow diagram.

This was a cross‐sectional study with a sample comprised of 133 children, which was the full sample of children born preterm, VLBW and admitted to the neonatal intensive care unit (NICU) of the Salamanca University Hospital between 2009 and 2011 (see Figure 1). The study was performed during the period of 2018–2020 (when children were between 7 and 10 years old) and was put on hold in February 2020 due to COVID‐19.

Recruitment procedure was established in two stages to ensure contact with the full sample. In the first stage, families from the selected sample were contacted via written letter. The letters enclosed information about the study, an informed consent agreement and a postage paid return envelope for families to return a signed copy of the informed consent, in the case that they agreed to collaborate with the research. Once the research team had received confirmation of participation, families were given an appointment to undergo initial assessment for the study via phone. In the second stage, families who did not respond to the letter were contacted by telephone to briefly explain the study and ask for collaboration. All parents who agreed to participate in the study were asked to sign the informed consent form before any evaluation took place.

### 2.2 Assessments and measures

#### 2.2.1 Demographics and postnatal factors

Demographics and postnatal information were collected from patien's clinical record from the NICU. A short questionnaire relating to demographic and personal information was also given to the parents.

#### 2.2.2 Assessments

Wechsler Intelligence Scale for Children–fifth edition (WISC–V)^19^ was used to measure cognitive development and the Autism Diagnostic Observation Schedule‐2 (ADOS‐2) module 3^20^ was applied to study ASD symptomatology and to support diagnostic evaluation. *Diagnostic and Statistical Manual of Mental Disorders‐5* criteria for ASD were used as a diagnostic gold standard.^14^

Positive cases on the ADOS‐2 were referred to both the diagnostic team from the research unit and the neuropaediatrician from the National Health System (NHS) of the Salamanca University Hospital to confirm the diagnosis.

### 2.3 Data analyses

Frequencies were estimated using Wilson score interval with continuity correction to compare the proportion of ASD in the sample to general prevalence rates. Kendall's *τb* correlation coefficient was calculated to see if there was any relationship between GA, BW, GP, and ASD diagnosis. A *τb* ≥ 0.13 (equivalent to a Pearson's *r* of 0.2) but <0.27 (Pearson's *r* of 0.4) was considered a weak association; ≥0.27 but <0.5 (Pearson's *r* of 0.7) as moderate; and a *τb* ≥ 0.5 as a strong association. Statistically significant results were evaluated at *p* < 0.05 and were onesided.

The sample was stratified based on the GA subcategories recommended by the World Health Organization (WHO).^21^ GP at birth were calculated with the GP calculator from the WHO^22^ using the GA in weeks and the birthweight in grams reported in the NICU records, alongside the children's sex.

Statistical analyses were performed using Statistical Package Social Sciences (SPSS) for Windows (version 23; IBM SPSS Statistics).

## 3 RESULTS

### 3.1 Sample characteristics and prevalence of ASD in the sample

A total of 133 children were included in the sample from the NICU of which 119 were eligible for collaboration in the study (see Figure 1). Of these, 64 agreed to participate, although only 57 completed full assessments, of which 4 children received a confirmed ASD diagnosis.

Mean age for the total sample at evaluation (*N* = 57) was 8.76 (SD = 0.86) and the mean intellectual quotient (IQ) was 98.19 (SD = 12.03). Of the four ASD cases, three (75%) were extremely preterm and in the lowest 2.5 GP (see Table 1). Non‐ASD cases were mostly very or late preterm (83%), 51% had GP lower than 2.5, and only a 13% were above median weight (GP ≥ 50).

**Table 1:** Sample characteristics.

	ASD cases	Non‐ASD cases
Gestational age (weeks)	*N* = 4 (%)	*N* = 53 (%)
24–27 (extremely preterm)	3 (75)	9 (17)
28–31 (very preterm)	1(25)	28 (53)
32–36 (moderate or late preterm)		16 (30)
Birthweight (g)	*N* = 4 (%)	*N* = 53 (%)
560–999	2 (50)	10 (19)
1000–1249	2 (50)	17 (32)
1250–1500		26 (49)
Growth percentiles for gestational age	*N* = 4 (%)	*N* = 53 (%)
Percentile < 2.5	3 (75)	27 (51)
Percentile ≥ 3–9		9 (17)
Percentile ≥ 10–49		10 (19)
Percentile ≥ 50–100	1 (25)	7 (13)
Sex	*N* = 4 (%)	*N* = 53 (%)
Male	3 (75)	24 (45)
Female	1 (25)	39 (55)
Assessment age (years)	*N* = 4 (%)	*N* = 53 (%)
7–8	1 (25)	21 (40)
9–10	3 (75)	32 (60)
IQ score	*N* = 4 (%)	*N* = 53 (%)
≤69		1 (2)
70–85		5 (9)
86–99		20 (38)
100–115	4 (100)	25 (47)
≥116		2 (4)

Abbreviations: ASD, autism spectrum disorder; IQ, intellectual quotient.

There was a 54% participation rate (*N* = 119 and 64 families willing to participate with the study, see Figure 1), giving a proportion of 7.02% (95% CI = 2.27%–17.83%) of ASD cases in this sample. A statistically significant weak correlation was found (*τb* = −0.23, *p* < 0.05) between GA and ASD diagnosis, suggesting a lower GA increases the likelihood of ASD diagnosis (see Table 2). Similarly, the correlation between ASD diagnosis and BW were statistically significant as well (*τb* = −0.25, *p* < 0.05), although the strong statistically significant association between GA and BW (*τb* = 0.57, *p* < 0.01) could mean they are confounding factors. Three of the four ASD cases had a GP < 2.5 and the other was the only case in the whole sample that had a GP ≥ 90. However, GP did not show a statistically significant correlation with ASD diagnosis in this sample (see Table 2).

**Table 2:** Kendall's *τb* correlations.

		GA (week)	BW (g)	GP
ASD diagnosis	*τb*	−0.23*	−0.25*	−0.05
	*p* (unilateral)	0.03	0.01	0.34
	*N*	57	57	57

Abbreviations: ASD, autism spectrum disorder; BW, birthweight; GA, gestational age; GP growth percentile.

* *p* < 0.05.

## 4 DISCUSSION

The main aim of this research was to study the preliminary results of ASD prevalence in preterm and VLBW children from the northwest of Spain. The estimated ASD prevalence for this sample was 7.02% (95% CI = 2.27%–17.83%), which, although the CI is quite large, is similar to the data shown in Agrawal et al.^15^ Other European studies reported a higher prevalence of 12.7% in a sample of very preterm children (*N* = 55) at the age of 3 years old^23^; or 11.9% in a cohort of extremely preterm children (*N* = 84) at 6.5 years old.^24^ Likewise, another study with preterm children showed a higher prevalence of 15.3% in a VLBW sample (*N* = 59) with an average age of diagnosis of approximately 4 years old.^25^

Additionally, the prevalence rate of ASD reported by the Centers for Disease Control and Prevention (2.3% or 1/44) is higher than those found in Europe. For example, a roughly 12‐fold increase is seen when the rate found in this study is compared to the 0.59% (95% CI = 0.48%–0.73%) population prevalence, reported by Fuentes et al.^26^ in a Spanish study; or similarly if European prevalence studies are taken as reference.^27^, ^28^, ^29^, ^30^ This highlights the increased likelihood of ASD for very preterm and VLBW children in Spain and even with a small sample size, these results are statistically significant.

Regarding GA and BW, both obtained a weak statistically significant association with ASD diagnosis. In a meta‐analysis that used risk ratios, both GA lower than 36 weeks and low BW were found to have significantly higher risk ratios for ASD.^18^ The current study found a statistically significant relationship between GA and ASD diagnosis, as well as between BW and ASD diagnosis, without reducing the dimensionality of the data. Overall, the current study suggests that treating GA and BW as continuous variables would allow for a better modeling of the relationship with ASD, although this would require a wider collaboration or data sharing to obtain sufficient data.

In relation to GP, no association was found. However, three of the ASD cases were SGA, while the other case was large for gestational age (LGA > 90th percentile), which was also the only LGA in the sample. This result is in line with other studies that have shown that both SGA and LGA have an increased risk of ASD, although the increased risk of LGA has normally been found in at‐term cases instead of preterm.^31^, ^32^ These results suggest that a nonlinear relationship may exist between GP and ASD.

### 4.1 Limitations and future research

As mentioned before, the number of participants made it difficult to obtain statistically significant results. As the VLBW criteria was quite restrictive, with only around 0.22% of births being under 1500 g, relaxing this criteria would lead to a larger sample size to evaluate. Participation in this study was adequate (54% of the 119 families which were contacted, see Figure 1), but there may be a participation bias as to why some families refused to collaborate (4%) or were unresponsive (20%).

Further research is needed with larger samples to generalize evidence shown in this study, but as this is first study in Spain which reports ASD prevalence data in a sample of preterm and VLBW children, it can be seen as an important step towards better understanding. The follow‐up of different cohorts over time would help to better describe ASD prevalence and characteristics in preterm children and support the development of specific early detection programs and interventions.

## 5 CONCLUSIONS

This is the first study in Spain which reports preliminary ASD prevalence data in a sample of preterm and VLBW children. The estimated prevalence for the sample was 7.02% (95% CI = of 2.27%–17.83%), suggesting a higher prevalence than the general population. These results support the latest meta‐analyses and studies regarding ASD for preterm children. Furthermore, using GA and BW as continuous variables was shown to be a potential method to help predict ASD diagnosis.

## AUTHOR CONTRIBUTIONS

**María Magán‐Maganto**: Conceptualization; data curation; formal analysis; investigation; methodology; project administration; writing—original draft; writing—review and editing. **Ricardo Canal‐Bedia**: Conceptualization; funding acquisition; investigation; project administration; resources; supervision; writing—original draft. **Álvaro Bejarano‐Martín**: Data curation; project administration; writing—original draft. **Maria Victoria Martín‐Cilleros**: Data curation; project administration. **Aránzazu Hernández‐Fabián**: Data curation; project administration. **Andrea Luz Calvarro‐Castañeda**: Data curation; project administration. **Herbert Roeyers**: Supervision; writing—original draft. **Cristina Jenaro‐Río**: Writing—original draft. **Manuel Posada de la Paz**: Methodology; supervision; writing—original draft. All authors have read and approved the final version of the manuscript.

## CONFLICT OF INTEREST STATEMENT

The authors declare no conflict of interest.

## ETHICS STATEMENT

All procedures performed in this study were in accordance with the ethical standards of the institutional research committee (Comité de Bioética de la Universidad, reference number: 201700031949, y Hospital de Salamanca, reference number: PI9910/2017) and with the 1964 Helsinki declaration and its later amendments or comparable ethical standards. Additional informed consents were obtained from all individual participants for whom identifying information is included in this article.

## TRANSPARENCY STATEMENT

María Magán‐Maganto affirms that this manuscript is an honest, accurate, and transparent account of the study being reported; that no important aspects of the study have been omitted; and that any discrepancies from the study as planned (and, if relevant, registered) have been explained.

## Data Availability

Anonymous data from this study is not included in any public repository. However, this data will be facilitated under request made to the first or corresponding author when the purpose is well described and justified. Ricardo Canal‐Bedia had full access to all of the data in this study and takes complete responsibility for the integrity of the data and the accuracy of the data analysis. The authors confirm that the data supporting the findings of this study are available within the article.

## References

[1] Howson CP , Kinney MV , McDougall L , Lawn JE , The Born Too Soon Preterm Birth Action Group . Born too soon: preterm birth matters. Reprod Health. 2013;10(suppl 1). 10.1186/1742-4755-10-S1-S1 PMC3828581 24625113

[2] Quinn JA , Munoz FM , Gonik B , et al. Preterm birth: case definition and guidelines for data collection, analysis, and presentation of immunisation safety data. Vaccine. 2016;34(49):6047‐6056. 10.1016/j.vaccine.2016.03.045 27743648 PMC5139808

[3] Instituto Nacional de Estadística Estadística de nacimientos . Datos definitivos. Resultados detallados. Año. 2020. Accessed June 29, 2022. https://www.ine.es

[4] Costeloe KL , Hennessy EM , Haider S , Stacey F , Marlow N , Draper ES . Short term outcomes after extreme preterm birth in England: comparison of two birth cohorts in 1995 and 2006 (the EPICure studies). BMJ. 2012;345:e7976. 10.1136/bmj.e7976 23212881 PMC3514472

[5] Twilhaar ES , de Kieviet JF , Aarnoudse‐Moens CS , van Elburg RM , Oosterlaan J . Academic performance of children born preterm: a meta‐analysis and meta‐regression. Arch Dis Child Fetal Neonatal Ed. 2018;103(4):F322‐F330. 10.1136/archdischild-2017-312916 28847871 PMC6047144

[6] Zerbeto AB , Cortelo FM , Filho ÉBC . Association between gestational age and birth weight on the language development of Brazilian children: a systematic review. J Pediatr (Rio J). 2015;91(4):326‐332. 10.1016/j.jpedp.2015.05.004 25913048

[7] World Health Organization . ICD‐10: International Statistical Classification of Diseases and Related Health Problems: Tenth Revision. World Health Organization; 2017.

[8] Cutland CL , Lackritz EM , Mallett‐Moore T , et al. Low birth weight: case definition & guidelines for data collection, analysis, and presentation of maternal immunization safety data. Vaccine. 2017;35(48 Pt A):6492‐6500. 10.1016/j.vaccine.2017.01.049 29150054 PMC5710991

[9] Schlaudecker EP , Munoz FM , Bardají A , et al. Small for gestational age: case definition & guidelines for data collection, analysis, and presentation of maternal immunization safety data. Vaccine. 2017;35(48 part A):6518‐6528. 10.1016/j.vaccine.2017.01.040 29150057 PMC5710996

[10] Easter SR , Eckert LO , Boghossian N , et al. Fetal growth restriction: case definition & guidelines for data collection, analysis, and presentation of immunization safety data. Vaccine. 2017;35(48 pt A):6546‐6554. 10.1016/j.vaccine.2017.01.042 29150060 PMC5710982

[11] Allotey J , Zamora J , Cheong‐See F , et al. Cognitive, motor, behavioural and academic performances of children born preterm: a meta‐analysis and systematic review involving 64 061 children. BJOG. 2018;125(1):16‐25. 10.1111/1471-0528.14832 29024294

[12] Sacchi C , Marino C , Nosarti C , Vieno A , Visentin S , Simonelli A . Association of intrauterine growth restriction and small for gestational age status with childhood cognitive outcomes: a systematic review and meta‐analysis. JAMA Pediatr. 2020;174(8):772‐781. 10.1001/jamapediatrics.2020.1097 32453414 PMC7251506

[13] Cordova EG , Belfort MB . Updates on assessment and monitoring of the postnatal growth of preterm infants. NeoReviews. 2020;21(2):e98‐e108. 10.1542/neo.21-2-e98 32005720

[14] American Psychiatric Association . Diagnostic and Statistical Manual of Mental Disorders (DSM‐5®). American Psychiatric Pub; 2013.

[15] Agrawal S , Rao SC , Bulsara MK , Patole SK . Prevalence of autism spectrum disorder in preterm infants: a meta‐analysis. Pediatrics. 2018;142(3):e20180134. 10.1542/peds.2018-0134 30076190

[16] Centers for Disease Control and Prevention (CDC) . Autism Spectrum Disorder (ASD). CDC; 2021 https://www.cdc.gov/ncbddd/autism/data.html

[17] Maenner MJ , Shaw KA , Bakian AV , et al. Prevalence and characteristics of autism spectrum disorder among children aged 8 years—autism and developmental disabilities monitoring network, 11 sites, United States, 2018. MMWR Surveill Summ. 2021;70(11):1‐16. 10.15585/mmwr.ss7011a1 PMC8639024 34855725

[18] Wang C , Geng H , Liu W , Zhang G . Prenatal, perinatal, and postnatal factors associated with autism: a meta‐analysis. Medicine. 2017;96(18):e6696. 10.1097/MD.0000000000006696 28471964 PMC5419910

[19] Lord C , Rutter M , DiLavore PC , Risi S , Gotham K , Bishop S . Autism Diagnostic Observation Schedule: ADOS‐2. Western Psychological Services; 2012.

[20] Wechsler D . Wechsler Intelligence Scale for Children®. 5th ed. Pearson; 2014.

[21] World Health Organization . Preterm Birth. World Health Organization; 2018. https://www.who.int/news-room/fact-sheets/detail/preterm-birth

[22] World Health Organization . Growth Percentiles Calculator. World Health Organization; 2020. http://srhr.org/fetalgrowthcalculator/#/

[23] Vermeirsch J , Verhaeghe L , Casaer A , Faes F , Oostra A , Roeyers H . Diagnosing autism spectrum disorder in toddlers born very preterm: estimated prevalence and usefulness of screeners and the Autism Diagnostic Observation Schedule (ADOS). J Autism Dev Disord. 2020;51(5):1508‐1527. 10.1007/s10803-020-04573-6 32757085

[24] Padilla N , Eklöf E , Mårtensson GE , Bölte S , Lagercrantz H , Ådén U . Poor brain growth in extremely preterm neonates long before the onset of autism spectrum disorder symptoms. Cereb Cortex. 2017;27(2):1245‐1252. 10.1093/cercor/bhv300 26689588

[25] Ikejiri K , Hosozawa M , Mitomo S , Tanaka K , Shimizu T . Reduced growth during early infancy in very low birthweight children with autism spectrum disorder. Early Hum Dev. 2016;98:23‐27. 10.1016/j.earlhumdev.2016.05.001 27367970

[26] Fuentes J , Basurko A , Isasa I , et al. The ASDEU autism prevalence study in northern Spain. Eur Child Adolesc Psychiatry. 2021;30(4):579‐589. 10.1007/s00787-020-01539-y 32388625

[27] Atladottir HO , Gyllenberg D , Langridge A , et al. The increasing prevalence of reported diagnoses of childhood psychiatric disorders: a descriptive multinational comparison. Eur Child Adolesc Psychiatry. 2015;24(2):173‐183. 10.1007/s00787-014-0553-8 24796725

[28] Boilson AM , Staines A , Ramirez A , Posada M , Sweeney MR . Operationalisation of the european protocol for autism prevalence (EPAP) for autism spectrum disorder prevalence measurement in Ireland. J Autism Dev Disord. 2016;46(9):3054‐3067. 10.1007/s10803-016-2837-y 27364514

[29] Delobel‐Ayoub M , Saemundsen E , Gissler M , et al. Prevalence of autism spectrum disorder in 7–9‐year‐old children in Denmark, Finland, France and Iceland: a population‐based registries approach within the ASDEU project. J Autism Dev Disord. 2020;50(3):949‐959. 10.1007/s10803-019-04328-y 31813107

[30] Narzisi A , Posada M , Barbieri F , et al. Prevalence of autism spectrum disorder in a large Italian catchment area: a school‐based population study within the ASDEU project. Epidemiol Psychiatr Sci. 2020;29:e5. 10.1017/S2045796018000483 PMC8061252 30187843

[31] Abel KM , Dalman C , Svensson AC , et al. Deviance in fetal growth and risk of autism spectrum disorder. Am J Psychiatry. 2013;170(4):391‐398. 10.1176/appi.ajp.2012.12040543 23545793

[32] Moore GS , Kneitel AW , Walker CK , Gilbert WM , Xing G . Autism risk in small‐and large‐for‐gestational‐age infants. Am J Obstet Gynecol. 2012;206(4):314.e1‐314.e9. 10.1016/j.ajog.2012.01.044 PMC9884028 22464070

